# Substantial improvement of tetraene macrolide production in *Streptomyces diastatochromogenes* by cumulative drug resistance mutations

**DOI:** 10.1371/journal.pone.0232927

**Published:** 2020-05-12

**Authors:** Jing-Xuan Fan, Yang Song, Gu Tang, Kozo Ochi, Xu-Ping Shentu, Xiao-Ping Yu

**Affiliations:** 1 Zhejiang Provincial Key Laboratory of Biometrology and Inspection & Quarantine, College of Life Sciences, China Jiliang University, Hangzhou, China; 2 Department of Life Science, Hiroshima Institute of Technology, Hiroshima, Japan; Universite Paris-Sud, FRANCE

## Abstract

Tetraene macrolides remain one of the most reliable fungicidal agents as resistance of fungal pathogens to these antibiotics is relatively rare. The modes of action and biosynthesis of polyene macrolides had been the focus of research over the past few years. However, few studies have been carried out on the overproduction of polyene macrolides. In the present study, cumulative drug-resistance mutation was used to obtain a quintuple mutant G5-59 with huge tetraene macrolide overproduction from the starting strain *Streptomyces diastatochromogenes* 1628. Through DNA sequence analysis, the mutation points in the genes of *rsmG*, *rpsL* and *rpoB* were identified. Additionally, the growth characteristic and expression level of *tetrRI* gene (belonging to the large ATP binding regulator of LuxR family) involved in the biosynthesis of tetraene macrolides were analyzed. As examined with 5L fermentor, the quintuple mutant G5-59 grew very well and the maximum productivity of tetramycin A, tetramycin P and tetrin B was as high as 1735, 2811 and 1500 mg/L, which was 8.7-, 16- and 25-fold higher than that of the wild-type strain 1628, respectively. The quintuple mutant G5-59 could be useful for further improvement of tetraene macrolides production at industrial level.

## Introduction

Polyene macrolide antibiotics are a large group of secondary metabolites and are potent antifungal agents with a unique mode of action that are currently being used in medicine and agriculture field [1, 2]. These antibiotics generally consist of a large macrolactone ring containing 25–37 carbon atoms, and deoxysugar mycosamine [1]. A series of four to eight alternating double bonds within the macrolactone ring are believed to be crucially important for the mode of action of polyenes [3, 4]. It is reported that all of tetraene macrolides have antifungal abilities, including more than 50 different members discovered since the early 1950s [5]. For example, natamycin has a broad-spectrum activity against yeasts and molds, with a low toxicity against mammalian cells [6], and is used as a safe food preservative by the Food and Drug Administration [7]. Likewise, tetramycin has been well used in controlling plant fungicidal diseases such as black spot diseases of forest trees [8]. Up to now, tetraene macrolides remain one of the most reliable fungicidal agents, as resistance of fungal pathogens to these antibiotics is relatively rare [4, 8]. Polyene macrolides are mainly synthesized by *streptomycetes* and related actinobacteria. Furthermore, polyene macrolides producers simultaneously synthesize several structurally related compounds [1, 9]. Although the chemical diversity of these metabolites is very important as a source of natural products, it may sometimes lead to an undesirable bottleneck for the industrial production of interesting compounds due to metabolic competition between them [9]. Therefore, activation of poorly expressed genes involved in the biosynthesis of polyene macrolide antibiotics by *Streptomyces* is very important in applied microbiology.

The “ribosome engineering” technology is an extremely effective method to increase activation of poorly expressed and cryptic genes in bacteria through the modulation of ribosomal components [10,11]. It has been applied on antibiotic overproduction and new active compound discovery in recent years because of its simplicity, wide applicability, and scalability for large-scale production [12–15]. This approach is based on the introduction of genetic mutations that confer resistance to drugs which target the ribosome, including streptomycin, gentamicin, paromomycin, neomycin, and others [12–15]. This ribosome engineering technology holds several advantages, i) the ability to screen for drug-resistance mutations through a simple selection that can be carried out on drug-containing plates even in the case of mutations with extremely low frequency (e.g., <10^−10^) and ii) the ability to select for mutations in the absence of known genetic information [16]. Interestingly, the introduction of several drug resistance mutations has a cumulative effect on antibiotic production [17, 18].

In our previous work, a strain *S*. *diastatochromogenes* (No.1628) was screened for biocontrol [17, 19]. Further studies demonstrated that this strain can produce at least 7 tetraene macrolides on the basis of ultraviolet absorption characteristics. Among them, 3 active components were separated and purified from strain 1628. These 3 antifungal components were identified as tetramycin A, tetramycin P (a new compound) and tetrin B using spectroscopic analysis including mass spectrometry and nuclear magnetic resonance spectroscopy [19, 20]. Recently, we have discovered that the acquisition of resistance to certain antibiotics, including rifampicin, paromomycin and streptomycin, improved the ability of *S*. *diastatochromogenes* 1628 to produce these three tetraene macrolides [19]. Thus, we demonstrate that the “ribosome engineering” is also effective in antibiotic overproduction by *S*. *diastatochromogenes* 1628.

In the current study, a *S*. *diastatochromogenes* 1628 mutant G5-59 with tetraene macrolides overproduction was obtained by cumulative drug-resistance mutation. The genes regulating the tetraene macrolides synthesis of 5 drug-resistant mutants and wild-type strain 1628 were investigated by real-time quantitative polymerase chain reaction (qPCR) technology. The mutation points of *rpsL*, *rsmG* and *rpoB* genes were detected in corresponding mutants. Finally, the growth characteristics of above 5 mutants and wild-type mutant were studied in 5-L fermentor.

## Materials and methods

### Strains

The wild-type strain *S*. *diastatochromogenes* 1628, deposited in the China General Microbiological Culture Collection (CGMCC) and assigned as accession number of CGMCC 2060, was isolated from soil sample [19]. The starting strain G1-99, which was single paromomycin-resistant mutant, was obtained during toyocamycin overproduction mutant screening and has higher tetraene macrolides productivity than the wildtype strain. The fermentation level of tetramycin A, tetramycin P and tetrin B of G1-99 strain was 2.1-, 3.4- and 2.9-fold of that produced by the wild-type strain 1628, respectively. Spontaneous rifampicin-resistant (Rif^r^), streptomycin-resistant (Str^r^), kanamycin-resistant (Kan^r^) and kasugamycin-resistant (Kas^r^) mutants were obtained from colonies that grew within 5 to 10 days after spore suspensions (~10^12^) were spread on GYM agar containing various concentrations of each respective antibiotic.

### Media and growth conditions

GYM medium was described previously [16]. A spore suspension of 0.5 ml (approximately 10^7^ spores per ml) was inoculated into 50 mL of the GYM medium and was incubated at 28 °C on a rotary shaker set to 200 rpm.

Three tetraene macrolides produced by *S*. *diastatochromogenes* 1628 and mutants were scaled up using a 5-L fermentor (BIOTECH-5BG, Baoxing Biological Equipment Co., Shanghai, China) containing 3 L of GYM medium. The spores of *S*. *diastatochromogenes* 1628 and its mutants (1 × 10^6^ spores/mL) were inoculated into 40 mL of seed medium 2XGYM [21] in a 250 mL Erlenmeyer flask at 28 °C, 180 rpm for 48 h. The agitation speed and aeration rate in fermentor were 250 rpm and 2 L/min, respectively. All assays were performed in triplicate.

### Determination of MICs

The minimum inhibitory concentrations (MICs) were determined by spotting spore suspensions (~10^6^) onto GYM plates containing various concentrations of a drug, followed by incubation at 28 °C for the indicated time. The minimum drug concentration able to fully inhibit growth was defined as the MIC.

### Analysis of tetraene macrolides and dry weight of biomass

Three tetraene macrolides were determined using a waters-e2695 HPLC (2998 PDA Detector, Waters Alliance Workstation, USA) with a column of RP-C_18_ column(250 mm×4.6 mm, 5 μm, XBridgeTM, Waters, USA). A water-CH_3_OH gradient system was used, which ranged linearly from 15% to 100% CH_3_OH over the course of 20 min, and was then held for 8 min. The detection wavelength was 304 nm and the solvent flow rate was 1.0 mL/min. The injection volume was 5 μL. The column temperature was maintained at 30 °C. Dry weight of biomass was described previously [17].

### Mutation analysis of rsmG, rpoB and rpsL

The *rsmG*, *rpoB* and *rpsL* genes were each amplified by PCR technology. The primers used to amplify candidate DNA fragments (*rsmG*, *rpoB* and *rpsL* genes) were designed as described previously [16, 21]. Purified PCR products were sequenced by Shanghai Sangni Biological Technology Co. The sequencing data were aligned using the Mega 6.0 program.

### Transcription analysis by real-time qPCR

Realtime quantitative PCR (qRT-PCR) was carried out using an Applied Biosystems StepOnePlus Real-Time PCR System with FastStar Universal SYBR Green Master (Rox) (Roche) [17]. The gene primers used in qRT-PCR reactions are listed in S1 Table. For each gene, all PCR reactions were carried out in triplicate within a single plate, with *rpoA* used as the reference gene. Quantification of relative gene expression was analyzed using the 2^-ΔΔCt^ method [22].

## Results

### Construction of combined drug-resistant mutants

Starting from the single-resistant mutant G1-99, we attempted to further enhance tetraene macrolides production by introducing drug-resistance mutations cumulatively. Double-resistant isolates were obtained by generating spontaneous mutants with high concentration streptomycin resistance. In total, 11 double mutants were screened and among of them, the mutant G2-13 exhibited best tetraene macrolides productivity. The fermentation level of tetramycin A, tetramycin P and tetrin B of G2-13 strain was 1.9-, 1.2- and 1.9-fold of that produced by the single-resistant mutant G1-99, respectively. Because no *rpsL* mutation was detected in any 11 double mutants, tetraene macrolides production was further improved by conferring resistance to higher concentration of streptomycin. Thirty one triple mutants were isolated, and three of which had greater ability to synthesize tetraene macrolides than the parental strain G2-13. The titer of tetramycin A, tetramycin P and tetrin B by G3-13 with the highest productivity among the triple mutant strains reached 390, 935 and 237 mg/L, which was 1.6-, 3.0- and 1.2-fold of that produced by the mutant G2-13 in GYM liquid medium (Table 1). Subsequent quadruple mutant screening was performed to isolate mutants that were resistant to higher concentration of streptomycin or kanamycin or kasugamycin. A total of 50, 18 and 53 spontaneous Str^r^, Kan^r^ and Kas^r^ were isolated, respectively. By HPLC analysis, it was found that the mutant G4-118 selected for higher-level resistance to streptomycin had an increased tetramycin A, tetramycin P and tetrin B production 1.9-, 1.7- and 5.2-fold, respectively (Table 1). The Kan^r^ G4-216 and Kas^r^ G4-316 had the highest productivity of 3 tetraene macrolides among 18 and 53 spontaneous Kan^r^ and Kas^r^ mutants, respectively. Neither of them was higher than Str^r^ G4-118 in terms of 3 tetraene macrolides productivity (Table 1). In order to introduce an *rpoB* mutation, which confers resistance to rifampicin, Rif^r^ strains were developed from the quadruple mutant G4-118. Only 2 Rif^r^ mutants were obtained and Rif^r^ mutant G5-59 produced 1033, 3011 and 768 mg/L tetramycin A, tetramycin P and tetrin B, more than 13-, 40- and 29-fold higher than that of the wild-type strain 1628 (Table 1). Another Rif^r^ mutant G5-36 had a reduced tetraene macrolides productivity.

**Table 1:** Screening and tetraene macrolides productivity of drug-resistant mutants.

Strain	Source	Antibiotic and concentration (mg /L) used for screening ^a^	Tetraene macrolides productivity (mg/L) ^b^		
			tetramycin A	tetramycin P	tetrin B
1628	-	-	60.6±3.2	73.1±2.6	36.6±1.8
G1-99	1628	Par(20)	126.3±8.3	250.6±10.1	104.8±3.2
G2-6	G1-99	Str(30)	200.9±9.8	287.9±15.4	165.9±10.8
G2-13	G1-99	Str(30)	244.7±13.5	310.6±16.8	201.5±9.2
G3-4	G2-13	Str(300)	298.6±18.1	653.7±29.8	274.6±11.4
G3-13	G2-13	Str(300)	390.5±15.2	935.8±20.2	237.3±13.5
G3-22	G2-13	Str(300)	353.8±9.8	789.5±18.9	221.3±9.9
G4-118	G3-13	Str(2500)	721.5±23.1	1557±48.6	1233.7±53.2
G4-216	G3-13	Kan(300)	450.1±16.6	976.4±30.2	664.1±33.0
G4-316	G3-13	Kas(1000)	546.3±25.7	1144.3±43.8	734.3±28.2
G5-36	G4-118	Rif(80)	269.6±19.9	714.8±23.0	363.2±17.6
G5-59	G4-118	Rif(80)	768.4±28.2	3011.6±68.9	1033.5±53.8

^a^ Numbers in parentheses show the used concentration.

^b^ Determined after 6 days of incubation at 28°C, 200 rpm, using a 300-mL flask containing 50 mL of GYM medium.

### Mutation analyses of the mutants

Antibiotic overproduction by ribosome engineering is attributed to certain mutations that alter the ribosome (i.e., *rsmG* and *rpsL* mutations) or RNA polymerase β-subunit (i.e., *rpoB* mutations) [23–28]. Therefore, we sequenced and compared the *rpsL*, *rsmG* and *rpoB* genes between mutants and wild-type strain. We found no mutations of the *rsmG* or *rpsL* gene in all 11 double mutants, though these mutants were obtained from the plates containing high level streptomycin and produced effective mutation. However, 3 triple-resistant mutants, all showing greater tetraene macrolides productivity than the starting strain G2-13, contained a mutation in the *rpsL* gene (Table 2). Mutants G3-4 and G3-22 had the same mutation point at nucleotide position 256, which is different from the nucleotide mutation position 124 in G3-13 mutant. Thus, the mutants G3-4 and G3-22 showed similar antibiotic productivity (Table 1). Among the quadruple mutants, there was no mutation in any of 3 gene(*rsmG*, *rpsL* and *rpoB*) of 18 Kan^r^ and 53 Kas^r^ mutants. As expected, an *rsmG* mutation was found at position 437 in quadruple mutant G4-118, which had strong tetraene macrolides productivity. Also as expected, both of quintuple mutants G5-36 and G5-59 with rifampicin resistance contained a mutation in the *rpoB* gene at position 1280 (Asp427→Val) and 1271 (Gln424→Leu).

**Table 2:** Strains and summary of mutations on *S*. *diastatochromogenes rpsL*, *rsmG* or *rpoB* gene resulting in amino acid exchange.

Strain ^a^	Genotype ^b^	Position in *rpsL* gene^c^	Amino acid substitution	Position in *rsmG* gene^c^	Amino acid substitution	Position in *rpoB* gene^c^	Amino acid substitution
1628	wild-type						
G1-99	*par*	ND	-	ND	-		
G2-6	*par str*	ND	-	ND	-		
G2-13	*par str*	ND	-	ND	-		
G3-4	*par str str*	256C→T	Arg86→Cys	ND	-		
G3-13	*par str str*	124C→T	Pro42→Ser	ND	-		
G3-22	*par str str*	256C→G	Arg86→Gly	ND	-		
G4-118	*par str str str*	124C→T	Pro42→Ser	437C→T	Ala146→Val		
G4-216	*par str str kan*	124C→T	Pro42→Ser	ND	-		
G4-316	*par str str kas*	124C→T	Pro42→Ser	ND	-		
G5-36	*par str str str rif*	124C→T	Pro42→Ser	437C→T	Ala146→Val	1280A→T	Asp427→Val
G5-59	*par str str str rif*	124C→T	Pro42→Ser	437C→T	Ala146→Val	1271A→T	Gln424→Leu

^a^ All mutant strains isolated in this study were spontaneous antibiotic-resistant mutants.

^b^ par: paromomycin, str: streptomycin, kan: kanamycin, kas: kasugamycin, rif: rifampicin.

^c^ Numbered in accordance with the numbering system for *S*. *coelicolor*.

ND: no mutation was detected.

### Growth characteristic of mutant strains

**Fig 1:**
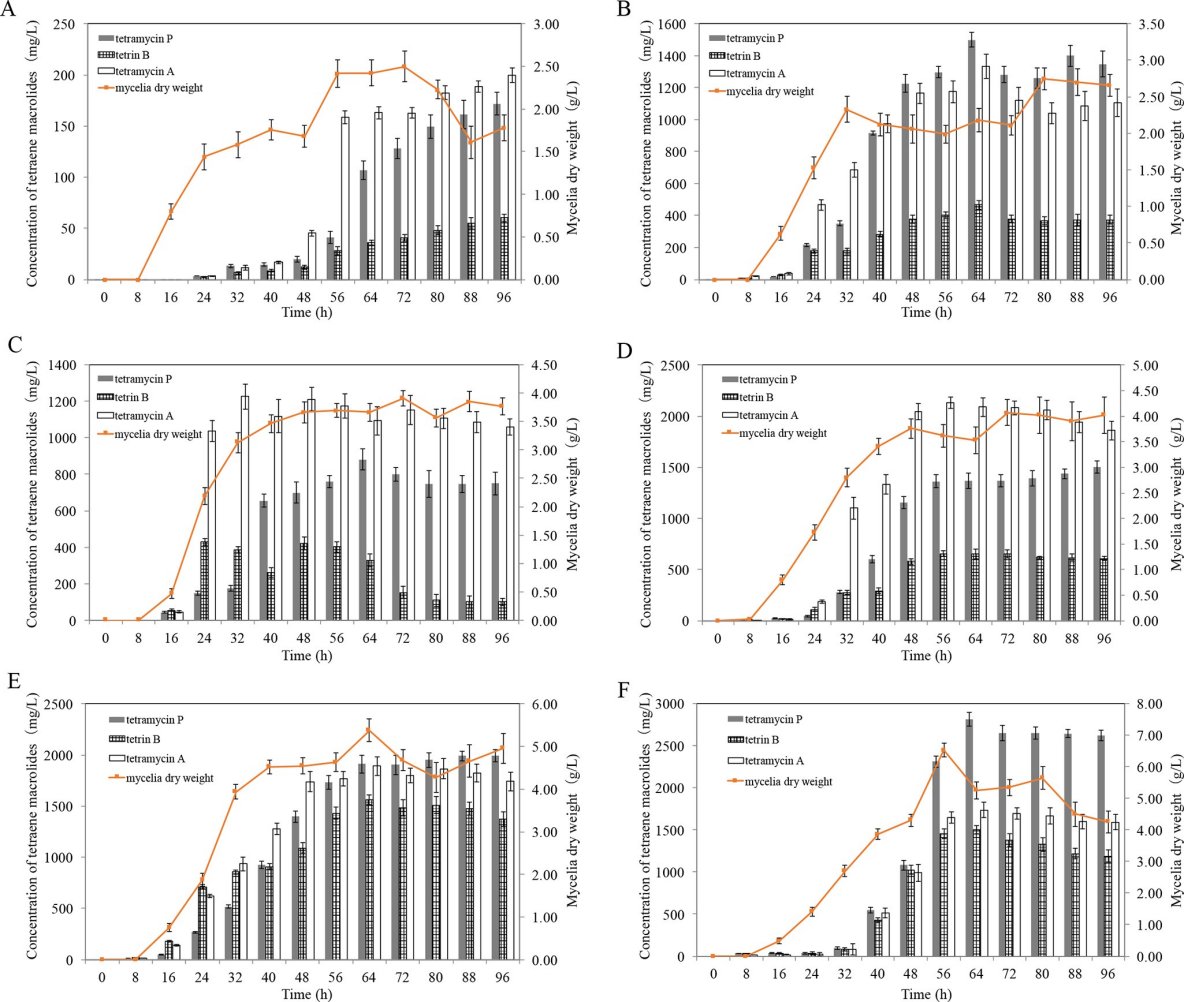
Comparison of tetraene macrolides production and cell growth in the parent and 5 mutant strains in the 5-L fermentor with GYM medium. **A.** wild strain 1628, **B.** single mutant G1-99, **C.** double mutant G2-13, **D**. triple mutant G3-13, **E**. quadruple mutant G4-118, **F**. quintuple mutant G5-59.

The introduction of drug-resistance by ribosome engineering often markedly changes the growth characteristic of mutants such as growth velocity in addition to antibiotic productivity [17, 18]. The batch fermentation experiments were scaled up using a 5-L fermentor to further investigate the growth characteristic of wild-type strain 1628 and mutant strains (G1-99, G2-13, G3-13, G4-118 and G5-59) (Fig 1A–1F). The growth of 6 strains entered logarithmic phase during 8 h to 56 h and stationary phase after 56 h. Surprisingly, all mutant strains grew as well as the wild-type strain in 5-L fermentor and produced tetraene macrolides even after entering into stationary-growth phase. At 8 h tetraene macrolides were detected in mutants, but until 24 h tetraene macrolides were not found in wild-type strain 1628, showing precocious production of antibiotics in mutants. Furthermore, 5 mutants showed much dense of hypha body and more bloom of growth in 5-L fermentor when compared with the wild-type strain 1628. For example, the maximal dry weights of G1-99, G2-13, G3-13, G4-118 and G5-59 reached 2.8, 4.2, 4.1, 5.4 and 6.5 g/L, respectively, which were 1.1-, 1.7-, 1.6-, 2.2- and 2.6-fold higher, respectively, than the wild-type strain.

Tetraene macrolides productivity in 5-L fermentor was essentially the same with flask fermentation, and all mutants produced more tetramycin A, tetramycin P and tetrin B than the wild-type strain 1628. The mutant strain G1-99 produced 1400 mg/L tetramycin P, 1333 mg/L tetramycin A and 470 mg/L tetrin B in 5-L fermentor, 8.2-, 7.8- and 3.6-fold higher, respectively, than the amount produced by wild-type and even more than double mutant strain G2-13 (Fig 1A–1C). The level of tetramycin P, tetrin B and tetramycin A produced by the triple mutant G3-13 had reached 1500, 660 and 2130 mg/L, an increase of 780%, 1000% and 1000% compared with wild-type 1628 (Fig 1D). The maximal yield of tetramycin P, tetrin B and tetramycin A in quadruple mutant G4-118 were all more than 1500 mg/L (1993, 1510 and 1900 mg/L, respectively) (Fig 1E). The quintuple mutant G5-59 could produce more tetramycin P compared with G4-118 and the yield was 2811 mg/L, which was 1.4-fold than the quadruple mutant. The tetrin B productivity (1510 mg/L) by the quintuple mutant G5-59 was almost equivalent with that of the G 4–118, while the productivity of tetramycin A decreased to some extent (Fig 1F).

### Relative expression level of TetrRI gene in wild-type and mutant strains

**Fig 2:**
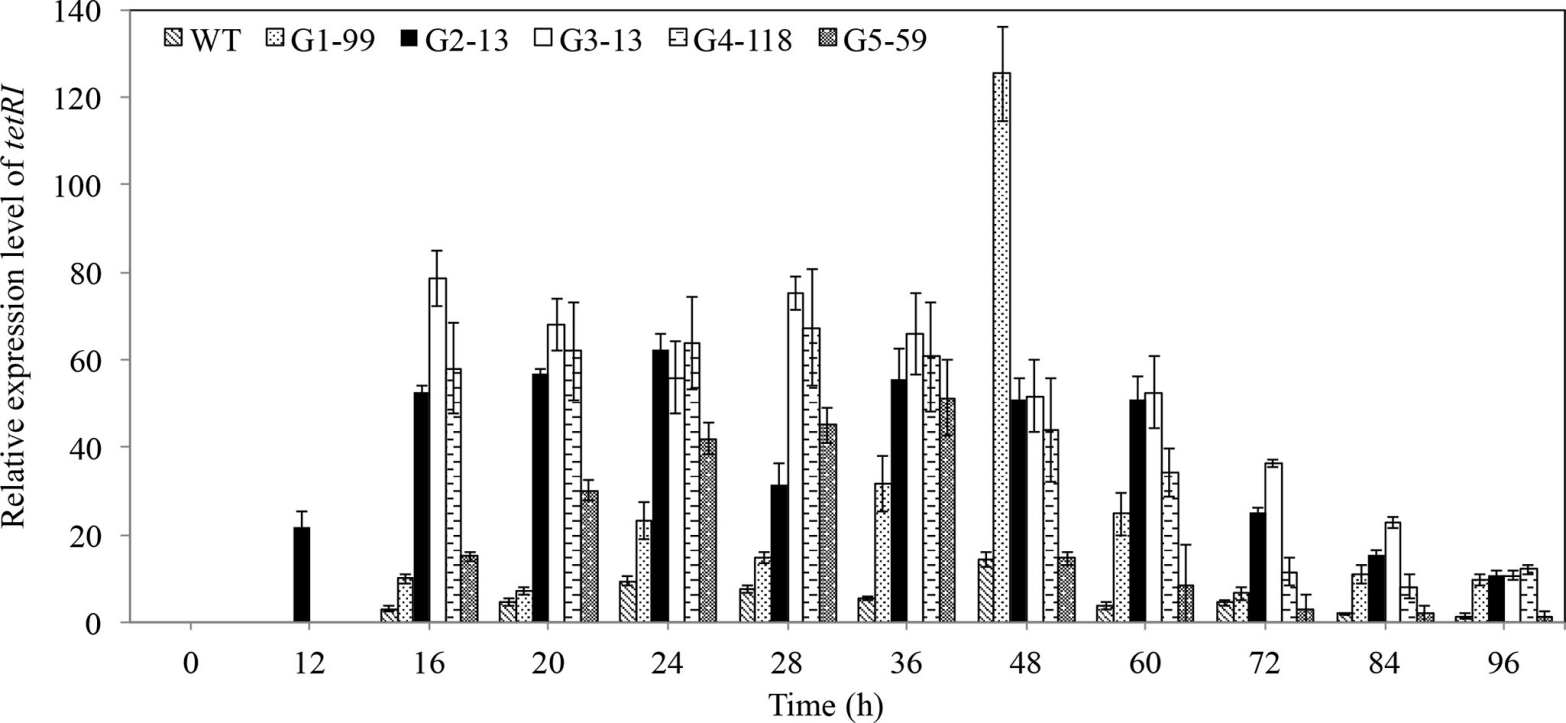
Relative expression level of *tetrRI* that acts as a large ATP binding regulator in tetraene macrolides biosynthesis. The expression level at 68 h in the wild type strain was defined as 1.

Up to now, the biosynthetic pathway of tetraene macrolides in *S*. *diastatochromogenes* remains unknown. It was reported that *tetrRI* gene, belonging to the LAL(large ATP binding regulator of LuxR) family, is involved in the biosynthetic pathway of tetraene macrolides in *S*. *hygrospinosus* [29]. Therefore, in order to clarify whether tetraene macrolides overproduction by mutants is attributed to the activated gene expression, transcription analysis of *tetrRI* gene in the wild-type and mutant strains (G1-99, G2-13, G3-13, G4-118 and G5-59) was performed using qRT-PCR technology. The expression levels of *tetrRI* gene in mutants were all higher than that of wild-type strain within the detection time (Fig 2). The expression of *tetrRI* mRNA was low in the wild-type strain 1628 throughout logarithmic and stationary growth phase. Strikingly, *tetrRI* expression showed a sharp increase in G1-99, G2-13, G3-13 and G4-118 mutant strains from 16 to 48 h. This period was also the rapid mycelium growth phase of these mutants. However, the quintuple mutant G5-59 showed lower *tetrRI* expression level. The maximal expression level of *tetrRI* in G1-99 (at 48 h), G2-13(at 24 h), G3-13 (at 28 h), G4-118 (at 28 h) and G5-59 (at 36 h) mutant strains were 8.9-, 4.4-, 5.4-, 4.8- and 3.7-fold compared with wild-type 1682 (at 48 h), respectively. Their maximum expression levels differed from each other, possibly accounting for the different productivity of tetramycin P, tetramycin A and tetrin B in mutants.

## Discussion

*S*. *diastatochromogenes* 1628 is an extremely valuable biological strain, isolated in our previous study [19], and able to synthesize at least 3 kinds of antibiotics, pyrrolopyrimidine nucleoside antibiotic-toyocamycin, tetraene macrolide antibiotics- tetramycin A, tetramycin P (TP, a novel compound) and tetrin B, and aromatic compound-anisomycin (not published). In a course to enhance the productivity of theses active constituents by strain 1628, efforts of sequential introduction of drug-resistance mutations substantially increased toyocamycin production in *S*. *diastatochromogenes* 1628. The triple mutant (SD3145) showed an enhanced capacity to produce toyocamycin (1500 mg/L), 24-fold higher than the wild-type strain in GYM medium [19]. High concentration of toyocamycin produced by toyocamycin overproduction mutant was always accompanied by low yield of tetraene macrolide antibiotics and anisomycin, perhaps because of substrate competition in metabolic system. In the present study, ribosome engineering technology was used to specifically enhance the tetraene macrolide antibiotics production by introducing quintuple mutations sequentially.

In the present study, 4 kinds of antibiotics (streptomycin, kanamycin, kasugamycin and rifampicin) were used as the screening drugs. The mechanisms underlying the generation of spontaneous streptomycin- and rifampicin-resistant mutants have been studied extensively in *Streptomyces* and *Bacillus* [15]. Mutants exhibiting high-level streptomycin resistance (≥20-fold MIC) often carried a mutation within *rpsL*, which encodes the ribosomal protein S12, and the mutation frequency was extremely low (e.g., <10^−10^) [27]. In contrast, mutants showing low-level streptomycin resistance (≥2-fold MIC) were found to have mutations in *rsmG* (rRNA small subunit methyltransferase G), which encodes a SAM-dependent 16S rRNA methyltransferase [30]. The *rsmG* mutation frequency was as high as 10^−6^ ~10^−4^, much higher than that of *rpsL* mutation. Low concentration of streptomycin was often used first in combination resistance screening. Tanaka et al. constructed *rpsL rsmG* double mutants by generating high-level streptomycin-resistant mutants from *rsmG* mutants [31]. Notably, production of the enzyme cycloisomaltooligosaccharide glucanotransferase (CITase) by *Paenibacillus agaridevorans* was enhanced dramatically by introducing *rsmG*, *rpsL*, and *rpoB* mutations successively [32]. In *S*. *diastatochromogenes*, *rsmG* mutation was successfully introduced into G3-13 mutant, which has a mutation in *rpsL* gene. Our results demonstrated that *rsmG* mutantion could be introduced into the high-level streptomycin resistant mutant with *rpsL* mutation.

It is well known that *rpoB* gene mutations, which arose in the RNA polymerase (RNAP) β-subunit, are responsible for the acquisition of rifampicin resistance and quite effective for activating the poorly expressed or silent secondary metabolite biosynthetic genes [23, 24]. Rifampicin resistance mutations are usually located in one specific conserved region of the Rif cluster within positions 1264C to 1327G, representing amino acid residues Leu422 to Ala443 [14]. It is reported that *rpoB* H437Y (His437 to Tyr) and H437R (His437 to Arg) mutations were most often effective in a wide variety of actinomycetes [16]. In the present study, the amino acid alterations Asp 427 to Val and Gln424 to Leu were found in the quintuple mutants G5-36 and G5-59, respectively, although causal relationship between these drug-resistance mutations and antibiotic overproduction was not studied in the present work.

The mechanisms underlying activation by *rpsL* and *rsmG* mutations have been studied extensively. Streptomycin-resistant *rpsL*-mutant ribosomes, which carry an amino acid substitution in ribosomal S12 protein that confers high-level resistance to streptomycin, are more stable than wild-type ribosomes, indicating that increased stability may enhance protein synthesis during the late growth phase of bacteria. Increased expression of the translation factor ribosome recycling factor also contributes to enhanced protein synthesis of the *rpsL* K88E mutant in late growth phase. That is, both the greater stability of the 70S ribosome and the elevated levels of ribosome recycling factor resulting from the *rpsL* K88E mutation are responsible for enhanced protein synthesis during late growth phase, with the latter being responsible for antibiotic overproduction and cryptic gene activation. Extensively increased biomass of drug-resistant mutants as observed in the present work could be accounted for by enhanced protein synthesis during late growth phase, although we did not determine protein synthesis activity in this work. In contrast, *rsmG* mutation of Streptomyces display markedly enhanced expression of SAM synthetase, activity of which was shown to be important in initiating antibiotic production, in that overexpression of *metK*, which encodes SAM synthetase, stimulates antibiotic production. These mechanisms are reviewed in detail in reference 15.

Ribosome engineering has been proved to be an effective approach for improving antibiotic productivity in actinomycetes [15]. Combinations of various drug-resistance mutations can further enhance bacterial productivity, as demonstrated, e.g., by introducing octuple drug-resistance mutations into *S*. *coelicolor* 1147 or triple drug-resistance mutations into *B*. *subtilis* [18, 33]. However, the octuple mutant C8 strain was somewhat temperature sensitive and showed extremely slow growth compared with wild-type strain 1147 [18]. In our previous work, we demonstrated that the triple mutant SD3145 produced a higher yield (1959 mg/L) of toyocamycin, which was 11-fold greater than that produced by the wild-type 1628. In addition, this mutant grew slowly [17]. In general, drug resistance is obtained accompanied by cost of growth fitness. Nevertheless, quintuple mutant G5-59 generated in the present work grew very well and the mycelia dry weight was even greater than that of wild-type 1628 as examined with 5-L fermentor. The *P*. *agaridevorans* triple mutant strain YT 478 also grew as well as the wild-type strain and produced CITase even after entering into stationary-growth phase [32]. These findings suggest that growth characteristic is dependent of strain, although mutant strains were introduced with different drug-resistance. Anyway, rapid growth of overproduction mutant strain is apparently advantageous for industrial application. The quintuple mutant strain G5-59, generated in the present study, could be useful for further improvement of tetraene macrolides production at industrial level.

## Supporting information

S1 Table Primers used for qRT–PCR. (DOC)
