# Supplementary material for: Substantial improvement of tetraene macrolide production in Streptomyces diastatochromogenes by cumulative drug resistance mutations
Source: PLoS One. 2020 May 12;15(5):e0232927. doi: 10.1371/journal.pone.0232927 (PMC7217443; doi:10.1371/journal.pone.0232927)
Supplement: S1 Table — (DOC) [file pone.0232927.s001.doc]

**S1 Table Primers used for qRT−PCR**

| **Gene name** | **Primer sequence(5’-3’)** |
| --- | --- |
| *tetrRI* F | TGACGCCGAAACGGAAA |
| *tetrRI* R | GCTGAGATACAGCCGAGAAG |
| *rpoA* F | GACGATCAGCTTGTCGAAGT |
| *rpoA* R | TACTCGCCGGTCCTCAA |
